# A survey of *Tmarus* Simon, 1875 (Araneae, Thomisidae) from Fanjing Mountain Nature Reserve, Guizhou, China

**DOI:** 10.3897/BDJ.11.e105352

**Published:** 2023-07-11

**Authors:** Weicheng Yang, Jinxiong Yang, Jianshuang Zhang, Hao Yu

**Affiliations:** 1 The State Key Laboratory of Southwest Karst Mountain Biodiversity Conservation of Forestry Administration, School of life sciences, Guizhou Normal University, Guiyang, China The State Key Laboratory of Southwest Karst Mountain Biodiversity Conservation of Forestry Administration, School of life sciences, Guizhou Normal University Guiyang China; 2 School of Biological Sciences, Guizhou Education University, Guiyang, China School of Biological Sciences, Guizhou Education University Guiyang China

## Abstract

**Background:**

*Tmarus* Simon, 1875 is a relatively large spider genus, currently includes 227 species distributed worldwide. Fanjing Mountain Nature Reserve is one of China’s most biodiverse regions. However, *Tmarus* can be regarded as being poorly represented in Fanjing Mountain, with only one species having been recorded so far: *T.fanjing* Yang & Yu, 2022.

**New information:**

Recently, various expeditions to Fanjing Mountain Nature Reserve were carried out by the authors. In this paper, two *Tmarus* species were brought to light by those expeditions: *T.fanjing* Yang & Yu, 2022 and *T.circinalis* Song & Chai, 1990. *T.fanjing* is redescribed, based on new material and the female is described and illustrated for the first time. The supplementary micrographs of *T.circinalis* are given for the first time. The DNA barcodes and a distribution map of both species are provided for future use.

## Introduction

*Tmarus* Simon, 1875 is the second most speciose genus of Thomisidae Sundevall, 1833, with 227 valid species distributed worldwide so far, after *Xysticus* C. L. Koch, 1835 (293 species), 27 species of which are recorded from China (World Spider Catalog 2023).

Although this genus is rather well known for its high species diversity, its taxonomy is very poorly studied: more than half of the species are known from a single sex or juveniles (five are described based on a juvenile; 143 species are known based on a single sex: for 53, only males are known, and for 90, only females are known) (World Spider Catalog 2023); for many species described in earlier studies, original descriptions are rather brief and lack illustrations or with inadequate illustrations (Tang and Li 2009, Yang et al. 2022).

Fanjing Mountain Nature Reserve, one of China’s most biodiverse regions, is located between 27°49’50”N to 28°01’30”N and 108°49’30”E to 108°18’30”E and is the core area of the Wuling Mountains (Qiu 2006). Fanjing Mountain as a representative of a primary forest ecosystem, famous for over 95% forest coverage (Guizhou Forestry Bureau 2014), known as ‘Earth Oasis’ or the ‘gene pool of animals and plants’. The only list of Fanjing Mountain spiders was published by Song et al. (2006). In this book, a total of 126 species belonging to 71 genera and 18 families were recorded. However, this estimate of spider diversity is assumed to be far from the true diversity within this Nature Reserve (Wang et al. 2015). Despite the fact that Thomisidae represents a substantial fraction of southwest China foliage-dwelling spiders (Tang and Li 2009, Tang and Li 2010), it can be regarded as being poorly represented in Mt. Fanjing, with only ten species from nine genera being clearly recorded: *Diaeasubdola* O. Pickard-Cambridge, 1885, *Ebrechtellatricuspidata* (Fabricius, 1775), *Lysitelesfanjingensis* Wang, Gan & Mi, 2020, *L.inflatus* Song & Chai, 1990, *Phartatangi* Wang, Mi & Peng, 2016, *Phrynarachnemammillata* Song, 1990, *Strigoplusguizhouensis* Song, 1990, *Tmarusfanjing* Yang & Yu, 2022, *Thomisuslabefactu*s Karsch, 1881 and *Xysticuskurilensis* Strand, 1907 (Song et al. 2006, Song and Chai 1990, Wang et al. 2016, Wang et al. 2020, Yang et al. 2022). Additionally, before *T.fanjing* described from Mt. Fanjing, no species of this genus have been reported from this region (Yang et al. 2022).

Recently various short, but intensive field collections in Fanjing Mountain have been conducted by staff of the Guizhou Normal University and Guizhou Education University. This paper reports our findings on the study of recently-available samples from the area, which revealed a new record species of Fanjing Mountain, *T.circinalis* Song & Chai, 1990, as well as the hitherto unknown female of *T.fanjing*. The aims of the current paper are: 1) to redescribe the male and report the female of *T.fanjing* for the first time; 2) to re-illustrate *T.circinalis*, based on new material from Mt. Fanjing and give supplementary micrographs; 3) to provide the DNA barcodes and a distribution map of *T.fanjing* and *T.circinalis* for future use.

## Materials and methods

Specimens in this study were collected by beating vegetation. Spiders were fixed and preserved in 95% ethanol. Specimens were examined with an Olympus SZX7 stereomicroscope; details were studied with an Olympus CX41 compound microscope. Female epigynes and male palps were examined and illustrated after being dissected. Epigynes were removed and cleared in warm lactic acid before illustration. The vulva was also imaged after being embedded in Arabic gum. Photos were made with a Cannon EOS70D digital camera mounted on an Olympus CX41 compound microscope. The digital images were taken and assembled using Helifocus 3.10.3. software package (Khmelik et al. 2006).

The distribution map was generated with ArcGIS v. 10.5 (Environmental Systems Research Institute, Inc.). Due to lack of locality coordinates in previous publications, locality coordinates for *T.circinalis* in Hubei Province and Chongqing City were originated from ArcGIS (see Song and Chai (1990)).

A DNA barcode was also obtained for the species matching. A partial fragment of the mitochondrial cytochrome oxidase subunit I (CO1) gene was amplified and sequenced for one male and one female specimen, respectively, using the primers LCOI1490 (5’-GGTCAACAAATCATAAAGATATTG-3’) and HCOI2198 (5’-TAAACTTCAGGGTGACCAAAAAAT-3’) (Folmer et al. 1994). For additional information on extraction, amplification and sequencing procedures, see Xu et al. (2022). Sequences were trimmed to 652 bp. All sequences were confirmed using BLAST and are deposited in GenBank. The codes and GenBank accession numbers of voucher specimen are provided as follows: *Tmarusfanjing*: YHTHO014, ♂, GenBank ON796486; YHTHO013, ♀, GenBank ON796487. *Tmaruscircinalis*: YHTHO015, ♂, GenBank OR075896; YHTHO016, ♀, GenBank OR075897.

All measurements were obtained using an Olympus SZX7 stereomicroscope and given in millimetres. Eye diameters are taken at the widest point. The total body length does not include chelicerae or spinnerets length. Leg lengths are given as total length (femur, patella, tibia + metatarsus, tarsus). Most of the terminologies used in text and figure legends follows Tang and Li (2009) and Zhang et al. (2022).

All specimens are deposited Museum of Guizhou Normal University, Guiyang, Guizhou, China.

## Taxon treatments

### Tmarus fanjing

Yang & Yu, 2022

42208F9D-B77B-5D32-BABA-3628DD1444C6

#### Materials

- **Type status:** Holotype. **Occurrence:** recordedBy: Da Wang; Jaiyuan Xin; individualID: YHTHO001; individualCount: 1; sex: 1 male; lifeStage: 1 adult; behavior: foraging; preparations: whole animal (ETOH); associatedSequences: GenBank: ON392063; occurrenceID: 7FC42291-EC27-5A7D-9D64-6E9F98746510; **Taxon:** order: Araneae; family: Thomisidae; genus: Tmarus; specificEpithet: *fanjing*; taxonRank: species; scientificNameAuthorship: Yang & Yu; taxonomicStatus: accepted; **Location:** continent: Asia; country: China; countryCode: CHN; stateProvince: Guizhou; county: Jiangkou; locality: Fanjingshan Nature Reserve; verbatimElevation: 755 m; decimalLatitude: 27.87; decimalLongitude: 108.80; **Identification:** identifiedBy: Jianshuang Zhang; dateIdentified: 12-12-2022; identificationReferences: Yang et al. 2022; **Event:** samplingProtocol: Beating; samplingEffort: 10 km by foot; year: 2021; month: 4; day: 18; **Record Level:** basisOfRecord: PreservedSpecimen
- **Type status:** Other material. **Occurrence:** recordedBy: Haonan Zhang; individualCount: 2; sex: 1 male, 1 female; lifeStage: 2 adults; behavior: foraging; preparations: whole animal (ETOH); occurrenceID: A38DE598-7685-5DA5-AB10-C63D1031394B; **Taxon:** order: Araneae; family: Thomisidae; genus: Tmarus; specificEpithet: *fanjing*; taxonRank: species; scientificNameAuthorship: Yang & Yu; taxonomicStatus: accepted; **Location:** continent: Asia; country: China; countryCode: CHN; stateProvince: Guizhou; county: Jiangkou; locality: Fanjingshan Nature Reserve; verbatimElevation: 1060 m; decimalLatitude: 27.90; decimalLongitude: 108.58; **Identification:** identifiedBy: Hao Yu; dateIdentified: 12-12-2022; identificationReferences: Yang et al. 2022; **Event:** samplingProtocol: Beating; samplingEffort: 10 km by foot; year: 2022; month: 7; day: 19; **Record Level:** basisOfRecord: PreservedSpecimen
- **Type status:** Other material. **Occurrence:** recordedBy: Cheng Wang; Xiaoqi Mi; individualID: YHTHO013, YHTHO014; individualCount: 4; sex: 2 males, 2 females; lifeStage: 4 adults; behavior: foraging; preparations: whole animal (ETOH); associatedSequences: ON796487; ON796486; occurrenceID: EE48AA39-2206-5DA9-8F4B-82BB2DA9B1D3; **Taxon:** order: Araneae; family: Thomisidae; genus: Tmarus; specificEpithet: *fanjing*; taxonRank: species; scientificNameAuthorship: Yang & Yu; taxonomicStatus: accepted; **Location:** continent: Asia; country: China; countryCode: CHN; stateProvince: Guizhou; county: Shiqian; locality: Fodingshan Nature Reserve; verbatimElevation: 858 m; decimalLatitude: 27.36; decimalLongitude: 108.0; **Identification:** identifiedBy: Cheng Wang; dateIdentified: 12-05-2022; identificationReferences: Yang et al. 2022; **Event:** samplingProtocol: Beating; samplingEffort: 10 km by foot; year: 2017; month: 4; day: 28; **Record Level:** basisOfRecord: PreservedSpecimen

#### Description

**Figure 1.**
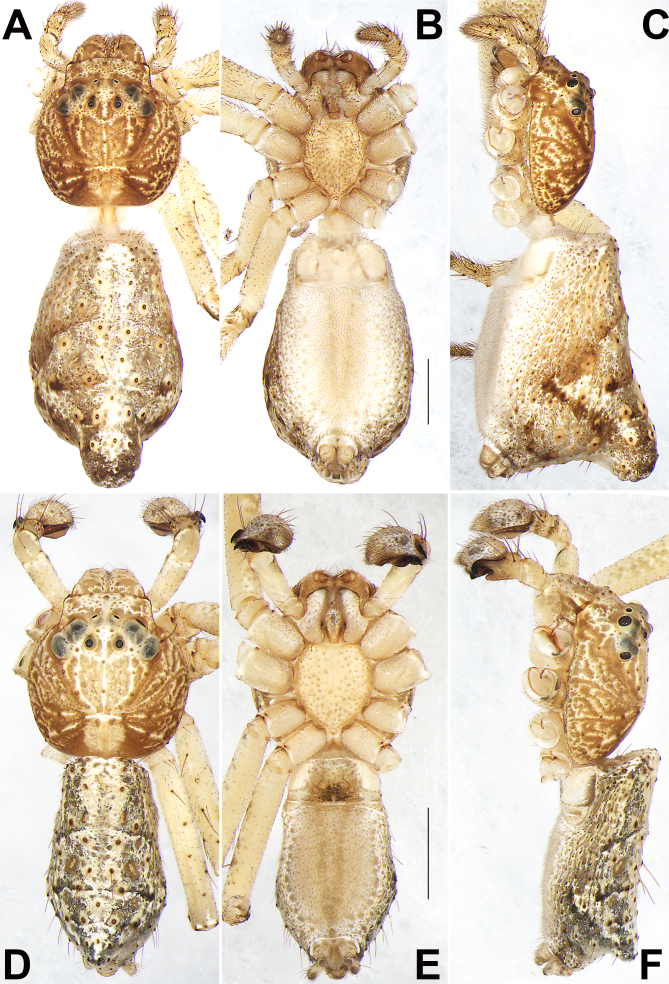
Habitus of *Tmarusfanjing*, female (**A–C**) and male (**D–F**). **A, D** Dorsal view; **B, E** Ventral view; **C, F** Lateral view. Scale bars: 1 mm (equal for A–C, equal for D–F).

**Figure 2.**
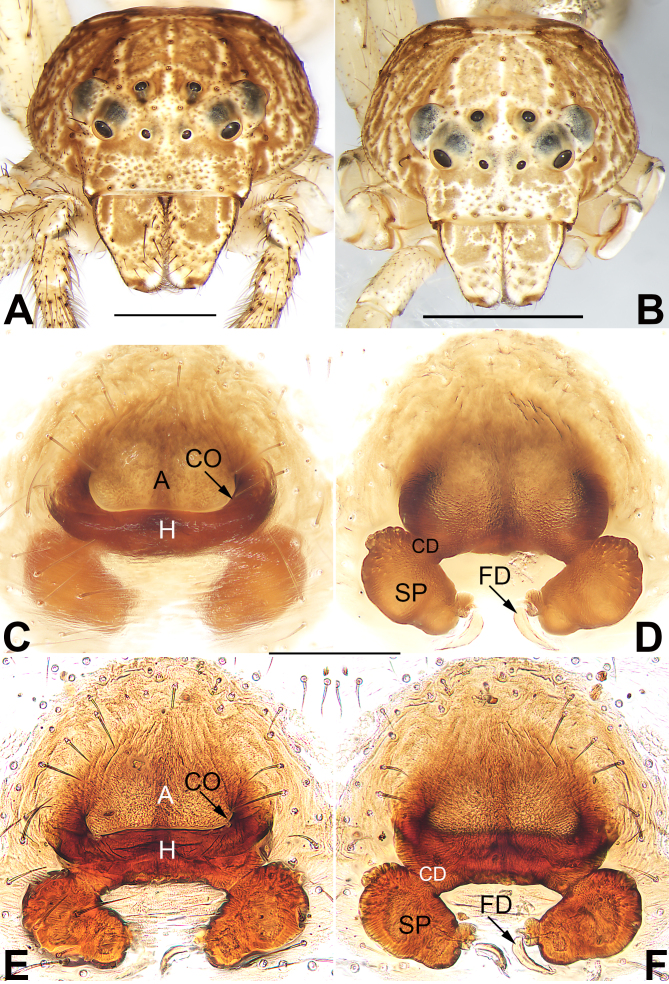
*Tmarusfanjing*, frontal views of prosoma (**A**–**B**) and epigyne (**C–F**). **A** Female; **B** Male; **C**–**D** Macerated epigyne, ventral and dorsal; **E**–**F** Epigyne, macerated and embedded in Arabic gum, ventral and dorsal. Abbreviations: A = atrium; CD = copulatory duct; CO = copulatory opening; FD = fertilisation duct; H = hood; SP = spermatheca. Scale bars: 1 mm (**A**–**B**), 0.2 mm (equal for **C–F**).

**Female** (Figs. 1A-C and 2A). Overall body colour is dull brown in ethanol. Total length 6.34; carapace 2.33 long, 2.25 wide; abdomen 4.01 long, 2.23 wide.

Carapace (Fig. 1A, C and Fig. 2A) dull brown mottled with light yellow and white patches, oval marking, light yellow outlines; ocular area slightly lighter; cervical groove and radial grooves distinguishable. In dorsal view, both anterior eye row (AER) and posterior eye row (PER) slightly recurved, PER distinctly wider than AER. Eye sizes and interdistances: anterior median eyes (AME) 0.08, anterior lateral eyes (ALE) 0.16, posterior median eyes (PME) 0.09, posterior lateral eyes (PLE) 0.14; distance between AMEs (AME–AME) 0.25, distance between AME and ALE (AME–ALE) 0.22, distance between PMEs (PME–PME) 0.34, distance between PME and PLE (PME–PLE) 0.49. Length of median ocular quadrangle (MOQ) 0.50, MOQ anterior width 0.39, MOQ posterior width 0.52. Clypeal height 0.42. *Chelicerae* coloured as ocular area, both margins without teeth. Labium and endites uniformly light brown, endites depressed posteriorly, slightly convergent anteriorly, with dense scopulae on anterior margin; labium nearly diamond-shaped, anterior margin with sparse setae. Sternum yellowish-white, more or less cordiform or shield-shaped, 1.20 long, 0.93 wide.

Abdomen (Fig. 1A–C) elongate, pyriform or shaped like a shield in dorsal view, tapering posteriorly, posteriorly with a prominent caudo-dorsal hump. Dorsum basically grey or light brown, with many long spiniform setae and greyish spots; venter yellowish-white, without distinct pattern; spinnerets brown.

Legs uniformly yellowish-white (Fig. 1A and B). Leg length: I 9.36 (2.88, 3.52, 1.95, 1.01), II 9.40 (2.85, 3.52, 2.00, 1.03), III 5.29 (1.75, 2.06, 0.87, 0.61), IV 5.57 (1.97, 2.03, 0.93, 0.64).

Epigyne (Fig. 2C–F). Epigynal plate slightly longer than wide, anterior and lateral margin not delimited, posterior margin rebordered; spermathecae (SP) clearly visible through the tegument in ventral view. Epigynal plate with an atrium (A) and a hood (H). Atrium large, represented by a deep depression, more than 2/3 epigyne width, anterior margin not rebordered, posterior margin delimited by the heavily sclerotised hood. Hood more or less ˽-shaped, nearly as wide as epigyne. Copulatory openings (CO) indistinct, at basolateral atrial borders, leading to copulatory ducts (CD) which descend obliquely to connect with spermathecae (SP). Spermathecae reniform, about 1.73 longer than wide, the two spermathecae separated by about one diameter. Fertilisation ducts (FD) acicular, membranous, located terminally on spermathecae.

**Male** (Fig. 1D–F and Fig. 2B). Total length 4.09; carapace 1.81 long, 1.75 wide; abdomen 2.28 long, 1.28 wide. Eye sizes and interdistances: AME 0.07, ALE 0.14, PME 0.09, PLE 0.12; AME–AME 0.13, AME–ALE 0.17, PME–PME 0.25, PME–PLE 0.32. MOQL 0.50, MOQA 0.30, MOQP 0.42. Sternum 0.93 long, 0.82 wide. Measurements of legs: I 11.1 (3.15, 4.00, 2.67, 1.28), II 10.95 (3.13, 3.94, 2.66, 1.22), III 5.24 (1.64, 2.02, 0.92, 0.66), IV 4.78 (1.76, 1.47, 0.95, 0.60). General characters as in female, but slightly smaller in size and lighter in colour.

**Figure 3.**
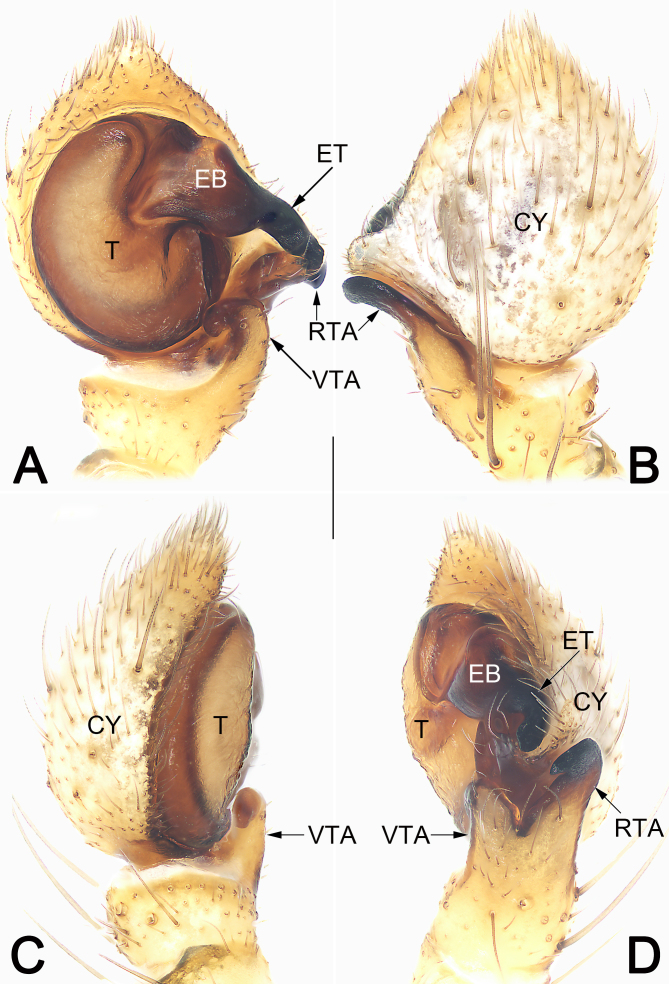
Male left palp of *Tmarusfanjing*. **A** Ventral view; **B** Dorsal view; **C** Prolateral view; **D** Retrolateral view. Abbreviations: CY = cymbium; EB = embolar base; ET = embolar tip; RTA = retrolateral tibial apophysis; T = tegulum; VTA = ventral tibial apophysis. Scale bar: 0.2 mm (equal for **A–D**).

Palp (Fig. 3A–D). Tibia relatively short, about 1/3 of cymbium length, with two apophyses: ventral apophysis (VTA) relatively short, nearly as long as tibia, apex blunt and curved, forming a semicircle in ventrally view; retrolateral apophysis (RTA) relatively long, ca. 1/2 of cymbium length, thumb-like in retrolateral view, apex rostrate and pointing ventrally. Tegulum (T) circular and relatively flat; sperm duct distinct, forming a loop along tegular margin. Embolus (E) thick and heavily sclerotised, finger-shaped; embolar base (EB) wide, partly membranous, inserted about 1~3 o’clock position of tegulum; embolar tip (ET), terminated at approximately 9 o’clock position relative to tegulum, apex slightly curved and blunt.

##### DNAbarcodes

5'TATTTGGAGCGTGATCGGCTATAGTAGGAACTGCTATAAGAGTATTGATTCGAATAGAATTAGGTAATTCAGGAAGACTTTTTGGAAATGATCATTTATATAATGTAATTGTGACTGCTCATGCTTTTGTGATAATTTTTTTTATAGTTATACCTATTTTAATTGGAGGATTTGGTAATTGATTAGTACCTTTGATATTAGGGGCTCCTGATATATCTTTTCCTCGAATAAATAATTTATCTTTTTGGTTATTACCTCCTTCTTTATTTTTATTATTTATATCTTCTATAGTAGAAATAGGAGTAGGAGCTGGATGAACTGTATATCCACCTTTGGCTTCTAGTTTAGGTCATATAGGGAGATCAATGGATTTTGCTATTTTTTCTCTTCATTTAGCTGGGGCTTCTTCAATTATAGGGGCTGTAAATTTTATTTCTACTATTATTAATATACGAAGAGTAGGAATGACTATAGAAAAGGTGCCTTTATTTGTCTGATCGGTGTTAATTACTGCTATTTTACTTTTATTATCATTACCTGTTTTAGCAGGAGCTATTACTATATTATTAACAGATCGAAATTTTAATACTTCGTTTTTTGACCCTGCTGGTGGAGGGGATCCAATTTTATTTCAACATTTATTTTGATTTTT3' (YHTHO013; Genebank accession number: ON796487).

5'TATTTGGAGCGTGATCGGCTATAGTAGGAACTGCTATAAGAGTATTGATTCGAATAGAATTAGGTAATTCAGGAAGACTTTTTGGAAATGATCATTTATATAATGTAATTGTGACTGCTCATGCTTTTGTGATAATTTTTTTTATAGTTATACCTATTTTAATTGGAGGATTTGGTAATTGATTAGTACCTTTGATATTAGGGGCTCCTGATATATCTTTTCCTCGAATAAATAATTTATCTTTTTGGTTATTACCTCCTTCTTTATTTTTATTATTTATATCTTCTATAGTAGAAATAGGAGTAGGAGCTGGATGAACTGTATATCCACCTTTGGCTTCTAGTTTAGGTCATATAGGGAGATCAATGGATTTTGCTATTTTTTCTCTTCATTTAGCTGGGGCTTCTTCAATTATAGGGGCTGTAAATTTTATTTCTACTATTATTAATATACGAAGAGTAGGAATGACTATAGAAAAGGTGCCTTTATTTGTCTGATCGGTGTTAATTACTGCTATTTTACTTTTATTATCATTACCTGTTTTAGCAGGAGCTATTACTATATTATTAACAGATCGAAATTTTAATACTTCGTTTTTTGACCCTGCTGGTGGAGGGGATCCAATTTTATTTCAACATTTATTTTGATTTTT3' (THO014; Genebank accession number: ON796486).

#### Diagnosis

Both sexes of *T.fanjing* are similar to those of *T.piger* (Walckenaer, 1802) (type species of *Tmarus*, see Ono (1977): 68, figs 1–6, 19, 28–31; Song and Zhu (1997): 51, fig. 28A–D) for the general shape of male palp and female vulva. *T.fanjing* and *T.piger* share the similar thick embolus directed obliquely and the large atrium (usually absent in many other *Tmarus* species). However, *T.fanjing* can be distinguished from *T.piger* by the following characters: for the males, embolus apex blunt in *T.fanjing* (vs. relatively sharp; Ono (1977): 68, figs 5, 6; Song and Zhu (1997): 51, fig. 28C); VTA apex curved, forming a semicircle in ventrally view (vs. not curved; Ono (1977): 68, figs 5, 6; Song and Zhu (1997): 51, fig. 28C); RTA nearly erect (vs. nearly horizontally directed; Ono (1977): 68, figs 5, 6; Song and Zhu (1997): 51, fig. 28C, D); for the females, epigyne ventrally with a hood in *T.fanjing* (vs. hood absent; Ono (1977): 68, fig. 3; Song and Zhu (1997): 51, fig. 28A).

#### Distribution

Known from the Mt. Fanjing and Mt. Foding, Guizhou Province, China (Fig. 7)

#### Taxon discussion

The species *Tmarusfanjing* Yang & Yu, 2022 was first described, based on male specimens only from Mt. Fanjing of Guizhou Province, China. Detailed description, diagnosis, high quality photographs and DNA barcoding of the holotype are provided in the original paper (see Yang et al. (2022)), to allow for easy species recognition. Recently, new materials containing both sexes were collected from the type locality and near the type locality (Mt. Foding, Guizhou Province, China; Fig. 7) simultaneously and seemed to be this species, based on comparison with the type specimen. DNA barcodes (a partial fragment of the mitochondrial cytochrome oxidase subunit I gene, COI) of the new materials was also obtained to confirm gender matching and species identification.

### Tmarus circinalis

Song & Chai, 1990

C26938E0-3415-5B9B-865F-B0E444B42836

#### Materials

- **Type status:** Other material. **Occurrence:** recordedBy: Da Wang; Jaiyuan Xin; individualID: YHTHO015, YHTHO016; individualCount: 5; sex: 2 males, 3 females; lifeStage: 5 adults; behavior: foraging; preparations: whole animal (ETOH); associatedSequences: GenBank: prepare to upload; occurrenceID: 9E9A0A7F-27B9-5732-81EE-36AD91E7A4E2; **Taxon:** order: Araneae; family: Thomisidae; genus: Tmarus; specificEpithet: *Tmaruscircinalis*; scientificNameAuthorship: Song & Chai; taxonomicStatus: accepted; **Location:** continent: Asia; country: China; countryCode: CHN; stateProvince: Guizhou; municipality: Jiangkou; locality: Fanjingshan Nature Reserve; verbatimElevation: 1025 m; decimalLatitude: 27.98; decimalLongitude: 108.69; **Identification:** identifiedBy: Hao Yu; dateIdentified: 15-01-2023; identificationReferences: Song and Zhu 1997; **Event:** samplingProtocol: Beating; samplingEffort: 10 km by foot; year: 2021; month: 7; day: 20; **Record Level:** basisOfRecord: PreservedSpecimen

#### Description

**Figure 4.**
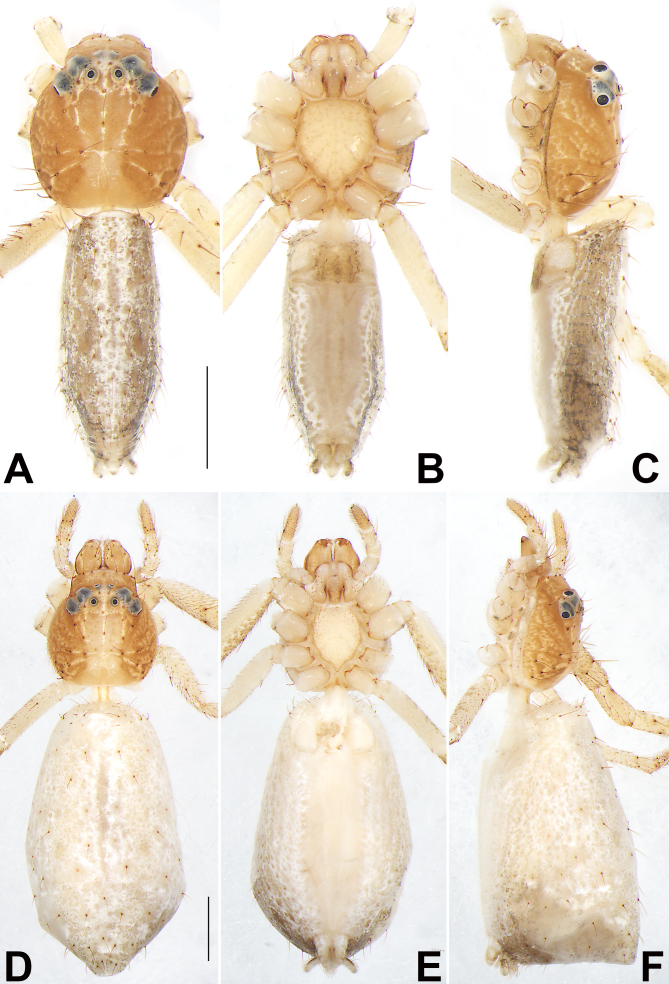
Habitus of *Tmaruscircinalis*, male (**A–C**) and female (**D–F**). **A, D** Dorsal view; **B, E** Ventral view; **C, F** Lateral view. Scale bars: 1 mm (equal for A–C, equal for D–F).

**Figure 5.**
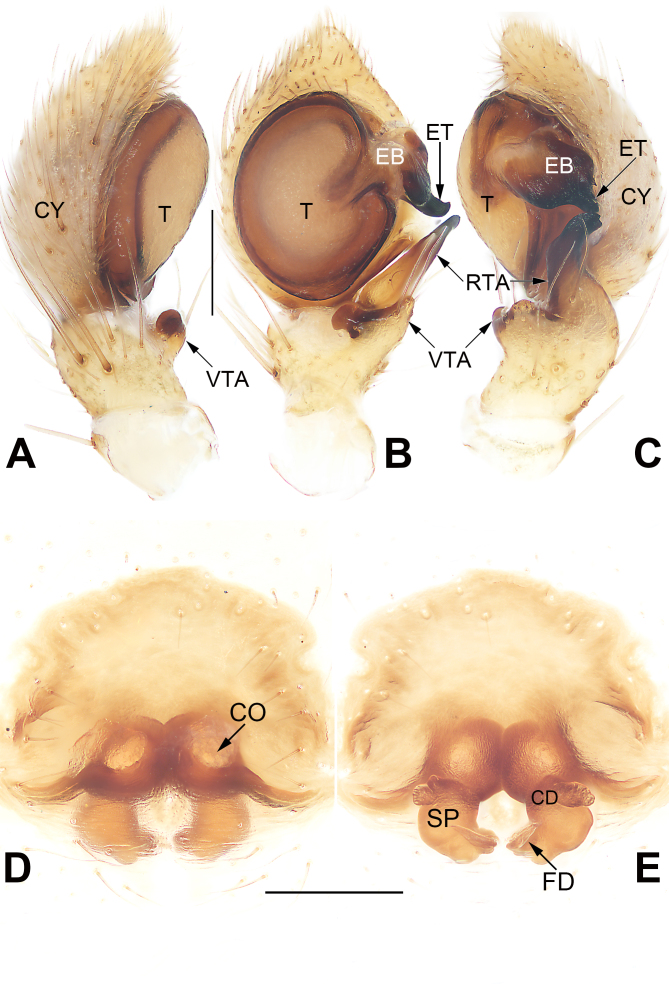
Male left palp (**A–C**) and female epigyne (**D–E**) of *Tmaruscircinalis*. **A** Prolateral view; **B** Ventral view; **C** Retrolateral view; **D–E** Macerated epigyne, ventral and dorsal. Abbreviations: CD = copulatory duct; CO = copulatory opening; CY = cymbium; EB = embolar base; ET = embolar tip; FD = fertilisation duct; RTA = retrolateral tibial apophysis; SP = spermatheca; T = tegulum; VTA = ventral tibial apophysis. Scale bar: 0.2 mm (equal for **A–D, D–E**).

**Figure 6.**
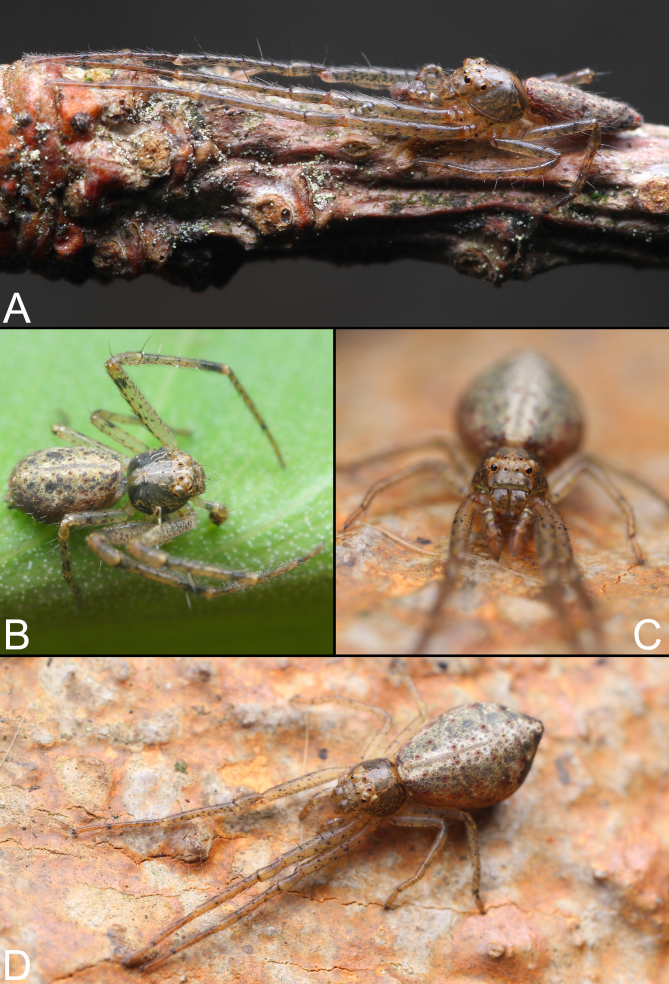
*Tmaruscircinalis*, male (**A–B**) and female (**C–D**), live specimens. Photographs by Qianle Lu (Shenzhen, Guangdong).

See Song and Chai (1990) and Song and Zhu (1997). Habitus as in Fig. 4, male palp as in Fig. 5A–C, epigyne as in Fig. 5D, E, live specimens as in Fig. 6.

##### DNAbarcodes

5'TATTTGGGGCGTGGTCAGCTATAGTAGGAACTGCTATAAGAGTATTAATTCGAATAGAATTGGGTAATTCAGGAAGACTTCTTGGTAATGATCATTTATATAATGTAATTGTGACTGCTCATGCTTTTGTAATAATTTTTTTTATAGTTATGCCTATTTTAATTGGAGGTTTTGGTAATTGATTAGTACCTTTGATATTAGGAGCTCCTGATATATCTTTTCCTCGAATAAATAATTTATCTTTTTGGTTATTACCTCCTTCTTTATTTTTATTATTTATATCTTCTATAGTGGAGATAGGAGTAGGGGCTGGGTGAACTGTATATCCACCTTTAGCTTCTAGTTTGGGTCATATAGGAAGATCAATGGATTTTGCTATTTTTTCTCTTCATTTAGCTGGGGCTTCTTCAATTATAGGGGCTGTAAATTTTATTACTACTATTATTAATATACGTAGAGTAGGAATAACTATAGAAAAAGTGCCTTTATTTGTTTGATCAGTGTTAATTACTGCTATTTTACTTTTACTATCATTACCTGTTTTAGCAGGAGCTATTACTATATTATTAACAGATCGAAATTTTAATACATCGTTTTTTGACCCTGCTGGAGGGGGGGATCCAATTTTATTTCAACATTTATTTTGATTTTT3' (YHTHO015; Genebank accession number: OR075896).

5'TATTTGGGGCGTGGTCAGCTATAGTAGGAACTGCTATAAGAGTATTAATTCGAATAGAATTGGGTAATTCAGGAAGACTTCTTGGTAATGATCATTTATATAATGTAATTGTGACTGCTCATGCTTTTGTAATAATTTTTTTTATAGTTATGCCTATTTTAATTGGAGGTTTTGGTAATTGATTAGTACCTTTGATATTGGGGGCTCCTGATATATCTTTTCCTCGAATAAATAATTTATCTTTTTGGTTATTACCTCCTTCTTTATTTTTATTATTTATATCTTCTATAGTGGAGATAGGAGTAGGGGCTGGGTGAACTGTGTATCCACCTTTAGCTTCTAGTTTGGGTCATATAGGGAGATCAATGGATTTTGCTATTTTTTCTCTTCATTTGGCTGGGGCTTCTTCAATTATAGGGGCTGTAAATTTTATTACTACTATTATTAATATACGTAGAGTAGGAATAACTATAGAAAAAGTGCCTTTATTTGTTTGATCAGTGTTAATTACTGCTATTTTACTTTTACTATCATTACCTGTTTTAGCAGGAGCTATTACTATATTATTGACAGATCGAAATTTTAATACATCGTTTTTTGACCCTGCTGGAGGGGGGGATCCAATTTTATTTCAACATTTATTTTGATTTTT3' (YHTHO016; Genebank accession number: OR075897).

#### Distribution

**Figure 7.**
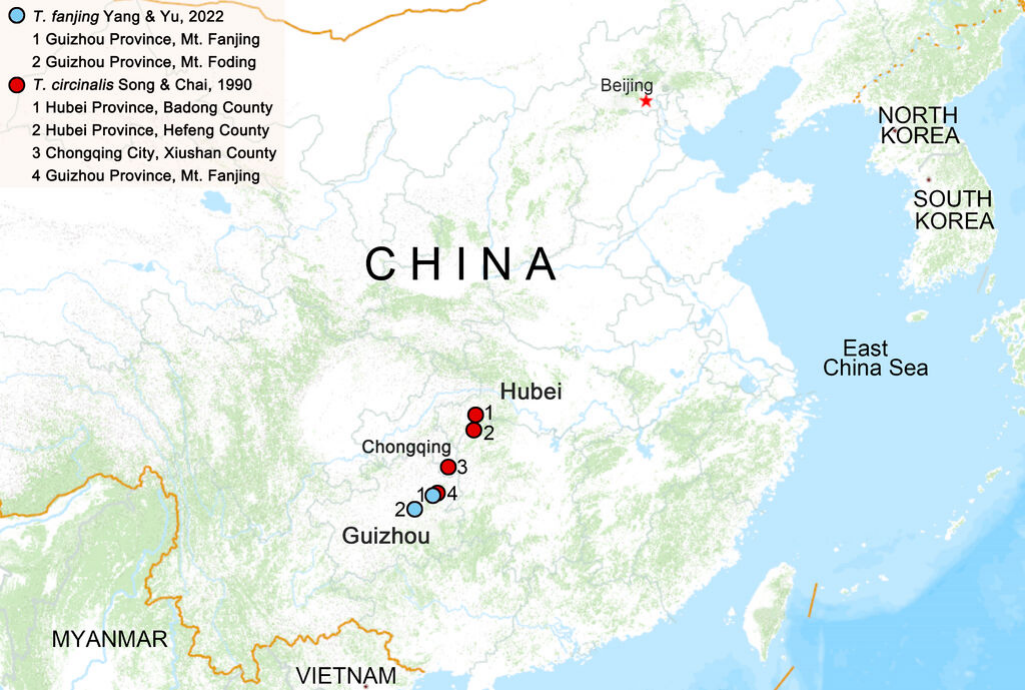
Distribution records of *Tmarusfanjing* and *Tmaruscircinalis*.

Hubei Province (Badong County and Hefeng County), Chongqing City (Xiushan County) and Guizhou Province (Mt. Fanjing), China (Fig. 7).

## Supplementary Material

XML Treatment for Tmarus fanjing

XML Treatment for Tmarus circinalis
